# Supplementary material for: Reconfiguration of interfacial energy band structure for high-performance inverted structure perovskite solar cells
Source: Nat Commun. 2019 Oct 9;10:4593. doi: 10.1038/s41467-019-12613-8 (PMC6785549; doi:10.1038/s41467-019-12613-8)
Supplement: Supplementary file 1 — Supplementary Information [file 41467_2019_12613_MOESM1_ESM.pdf]

Supplementary information

Reconfiguration of interfacial energy band structure for high-performance inverted structure perovskite solar cells

Zhang et al.

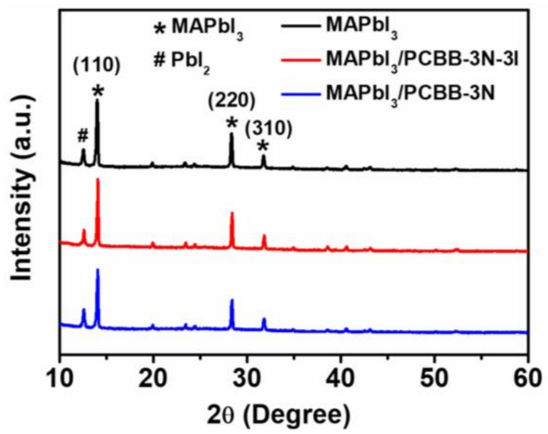

Supplementary Figure 1. XRD spectra of $MAPbI_3$ before and after PCBB-3N-3I/PCBB-3N treatment.

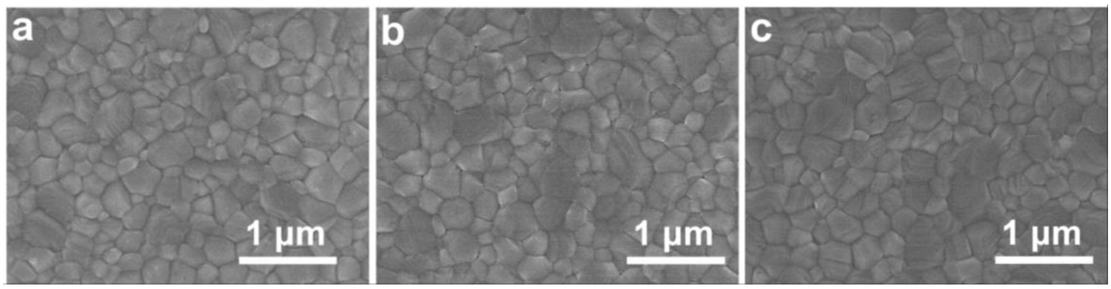

Supplementary Figure 2. SEM images of $MAPbI_3$ (a), $MAPbI_3$/PCBB-3N-3I (b) and $MAPbI_3$/PCBB-3N (c) films.

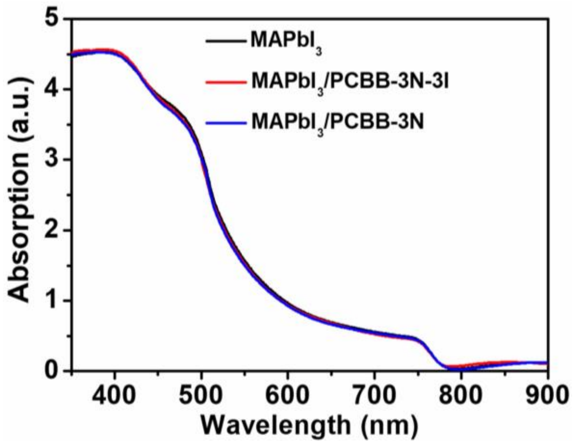

Supplementary Figure 3. Absorption spectra of $MAPbI_3$ before and after PCBB-3N-3I/PCBB-3N treatment.

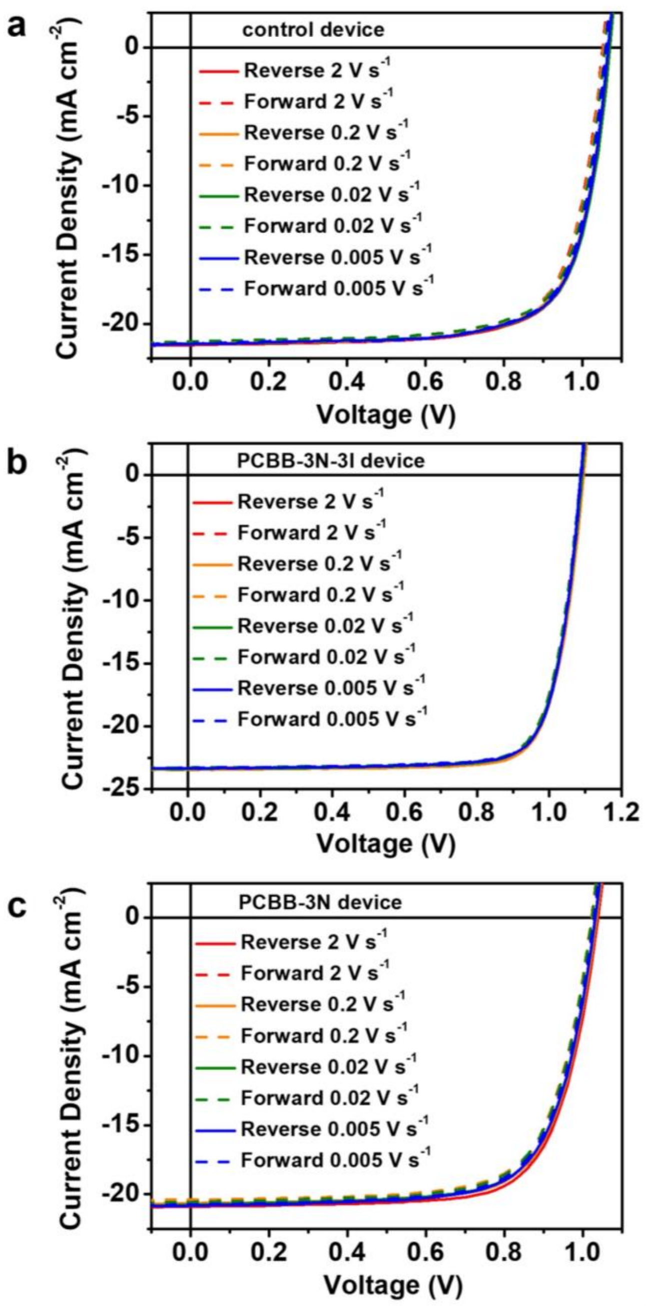

Supplementary Figure 4. *J–V* curves of control device (a), PCBB-3N device (b) and PCBB-3N-3I device (c) under AM 1.5G illumination at scan rates of 0.005, 0.02, 0.2, 2.0 V s^{-1} for both forward (0 to 1.2 V) and reverse scanning (1.2 to 0 V).

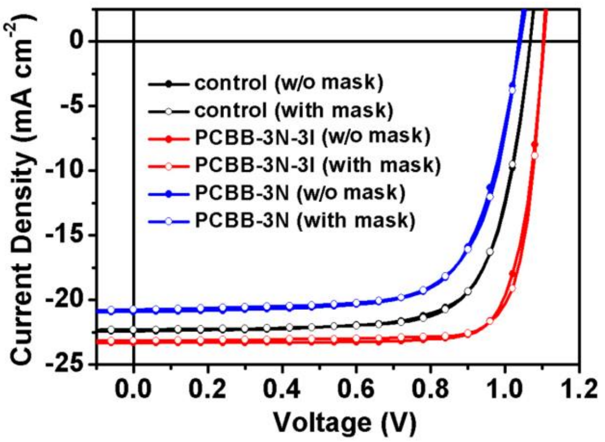

Supplementary Figure 5. *J*-*V* curves of control device, PCBB-3N device and PCBB-3N-3I device under AM 1.5G illumination with 0.049 cm^2 mask or w/o mask.

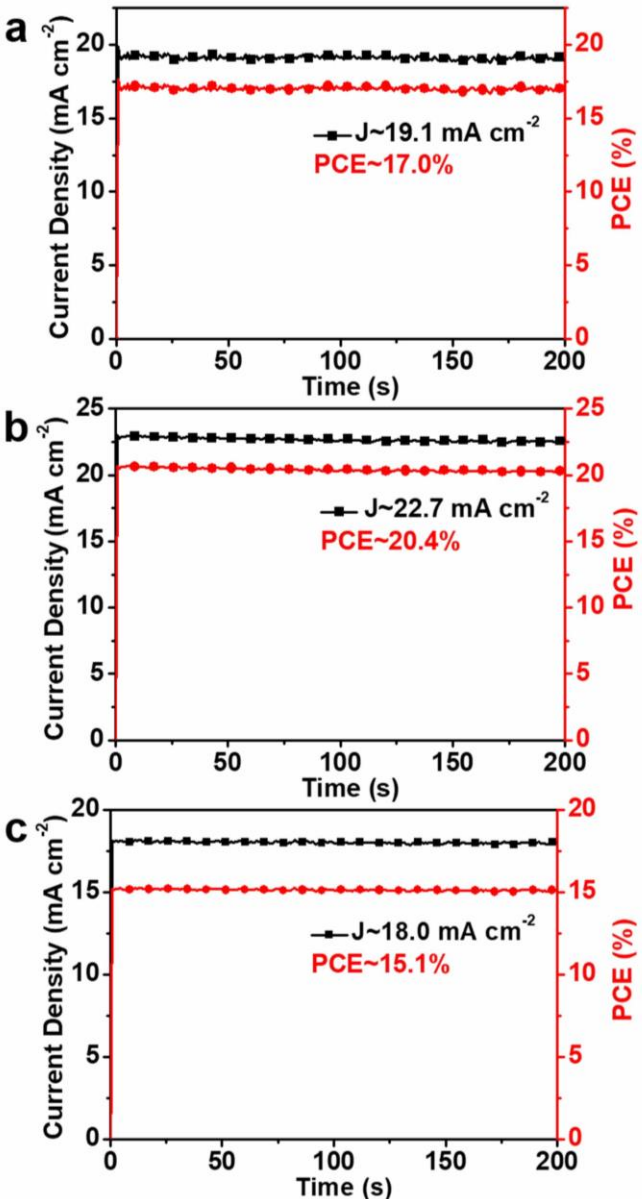

Supplementary Figure 6. The stabilized PCEs at MPP for control device (a), PCBB-3N-3I device (b) and PCBB-3N device (c).

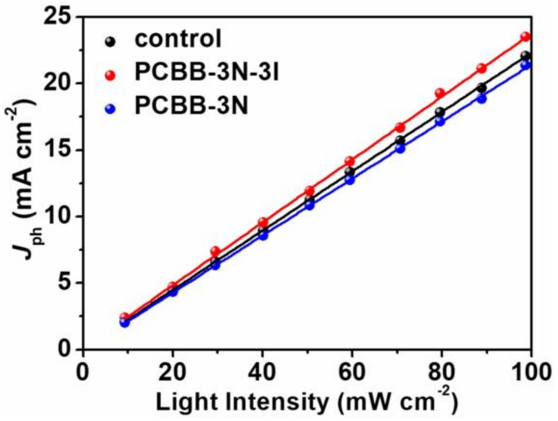

Supplementary Figure 7. Light intensity dependent J_{ph} for control device, PCBB-3N-3I device and PCBB-3N device.

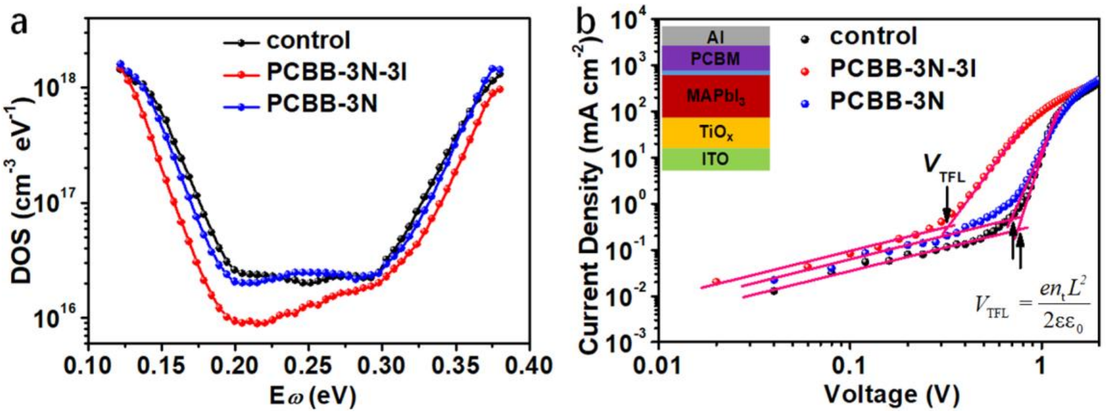

Supplementary Figure 8. Trap density measurements. (a) Trap density of states (tDoS) of control device, PCBB-3N-3I device and PCBB-3N device extracted from thermal admittance spectroscopy. (b) *J*-*V* curves of electron-only devices with a structure of ITO/TiO_x/$MAPbI_3$/PCBM/Al with and w/o PCBB-3N-3I/PCBB-3N treatment. The smaller trap-filled limited voltage (V_{TFL}) with PCBB-3N-3I treatment indicates reduced trap density n_t, which agrees with lower tDOS in PCBB-3N-3I device. The little change in V_{TFL} with PCBB-3N treatment means comparable n_t, which supports little difference in tDoS between PCBB-3N-3I device and control device.

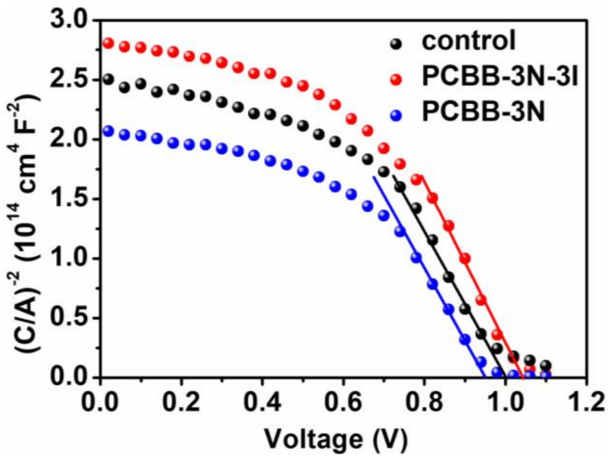

Supplementary Figure 9. Mott–Schottky plots of the pero-SCs before and after PCBB-3N-3I/PCBB-3N treatment.

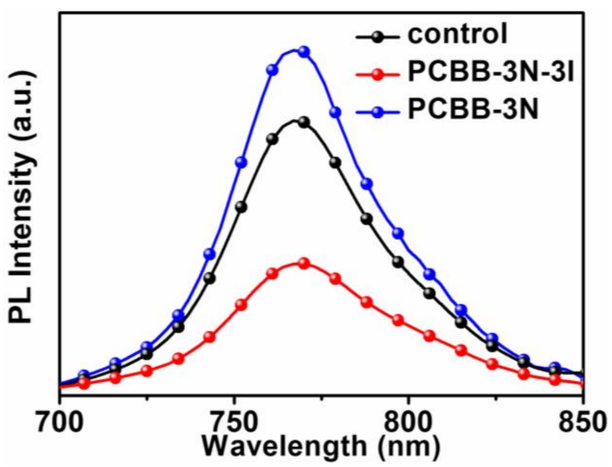

Supplementary Figure 10. PL spectra of $MAPbI_3$/PCBM on glass substrate before and after PCBB-3N-3I/PCBB-3N treatment.

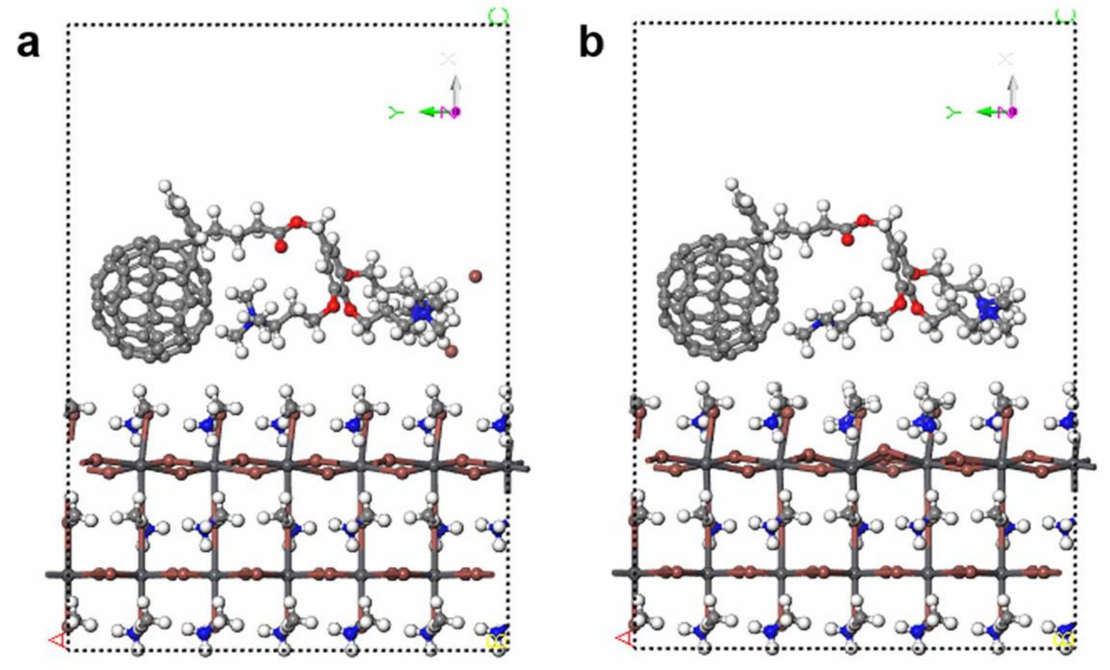

Supplementary Figure 11. Configurations of PCBB-3N-3I (a) and PCBB-3N (b) on $MAPbI_3$ for DFT calculation.

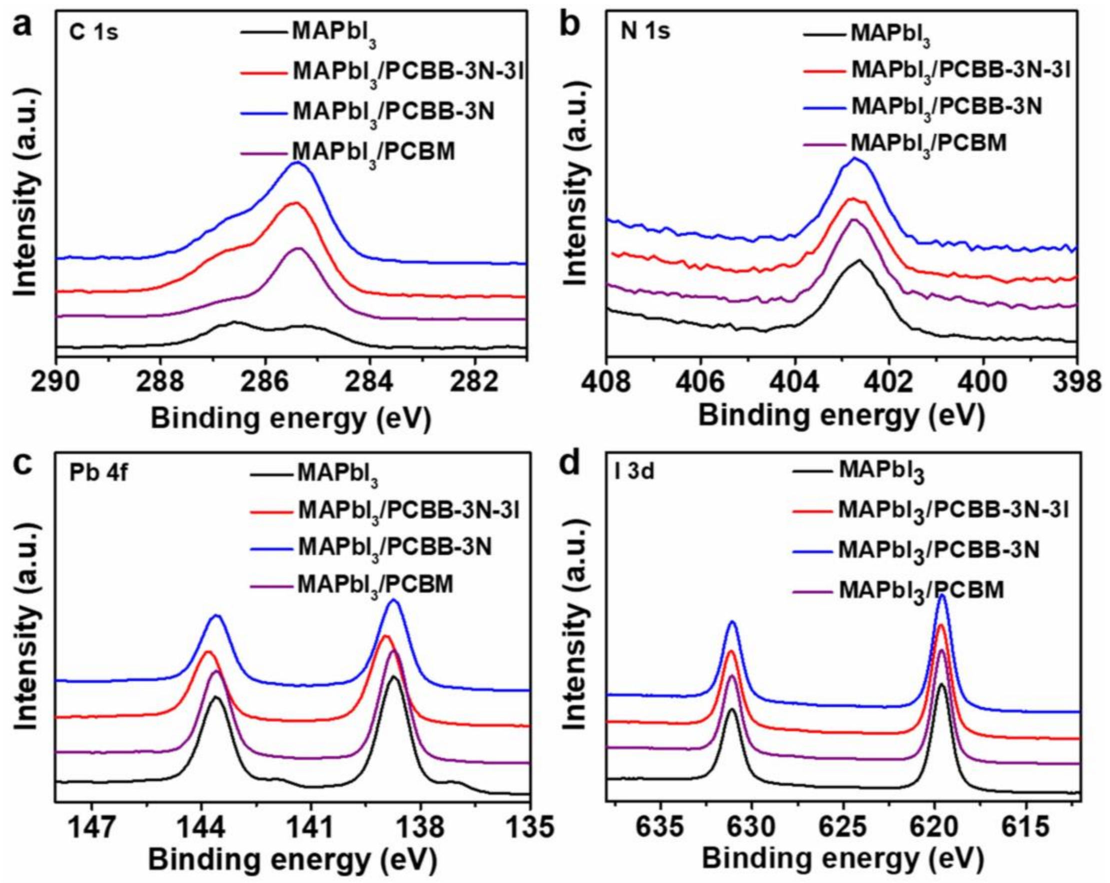

Supplementary Figure 12. XPS for C 1s (a), N 1s (b), Pb 4f (c), I 3d (d) of $MAPbI_3$ before and after PCBB-3N-3I/PCBB-3N treatment.

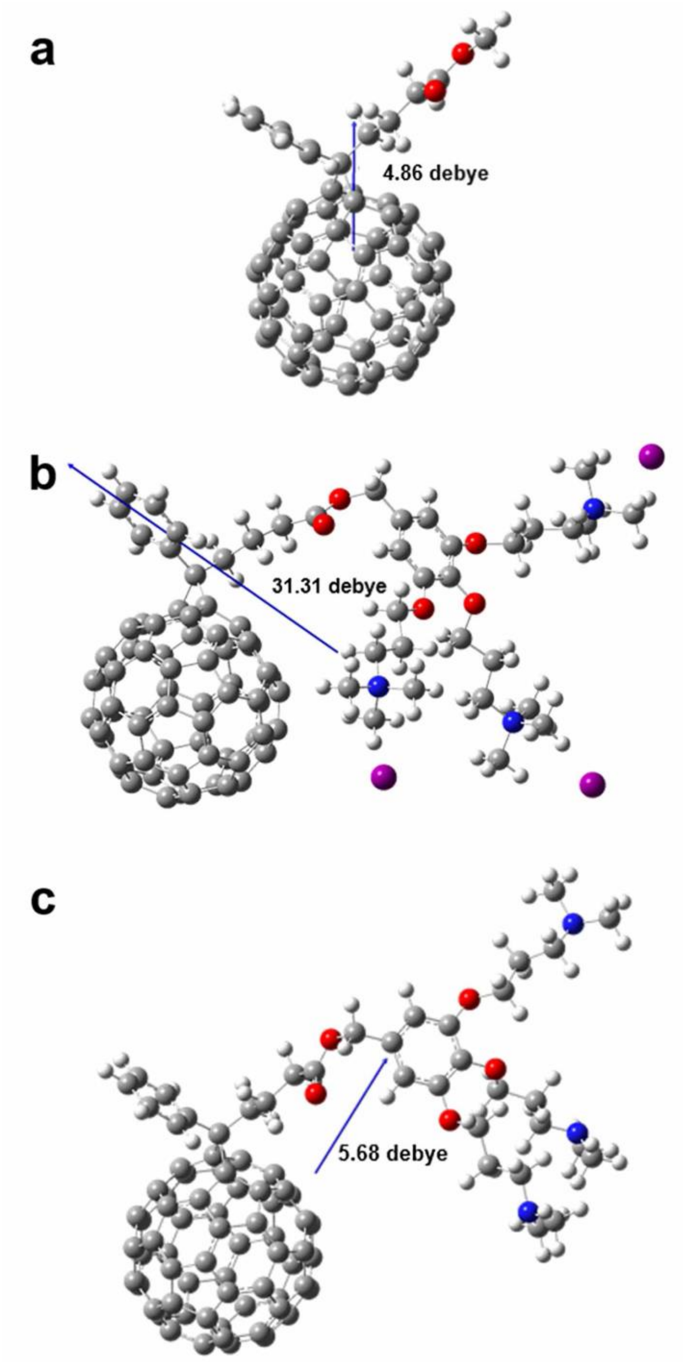

Supplementary Figure 13. Molecular electric dipole moment of PCBM (a), PCBB-3N-3I (b), PCBB-3N (c) based on DFT calculation.

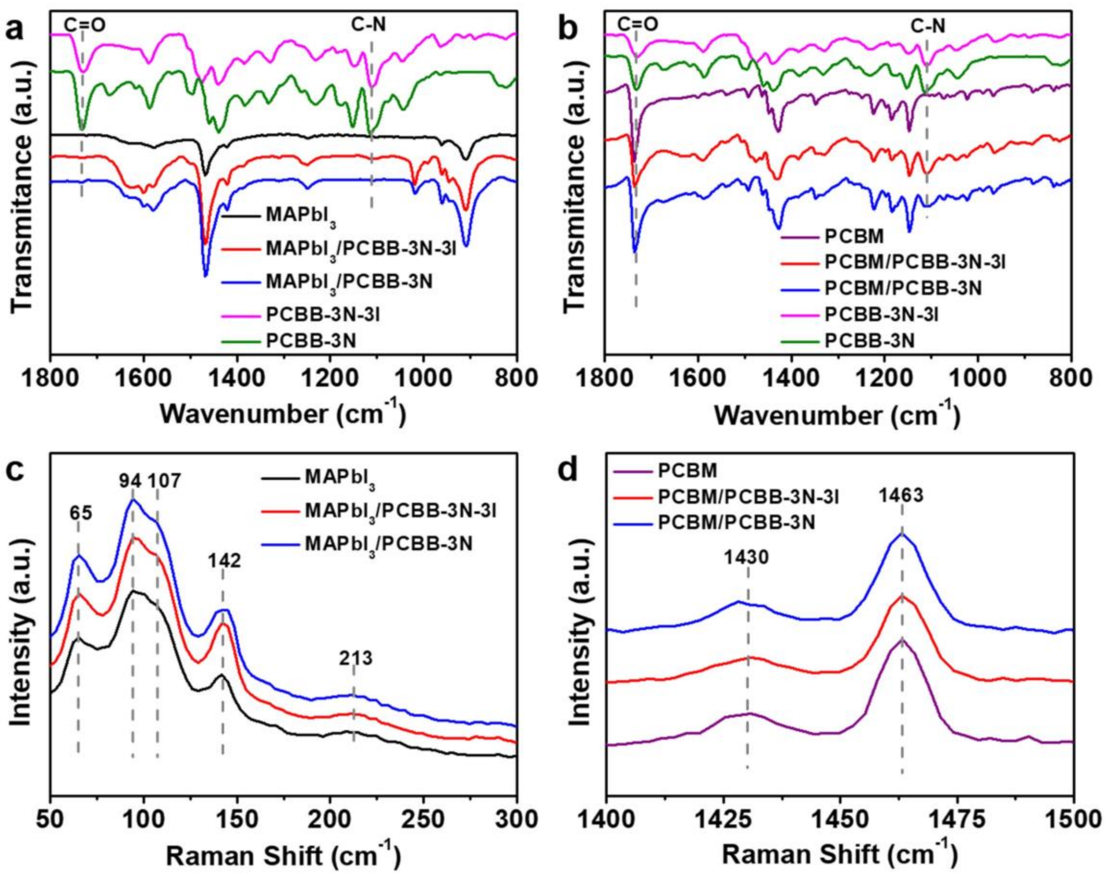

Supplementary Figure 14. FT-IR (a-b) and Raman (c-d) spectra of $MAPbI_3$ and PCBM before and after PCBB-3N-3I/PCBB-3N treatment.

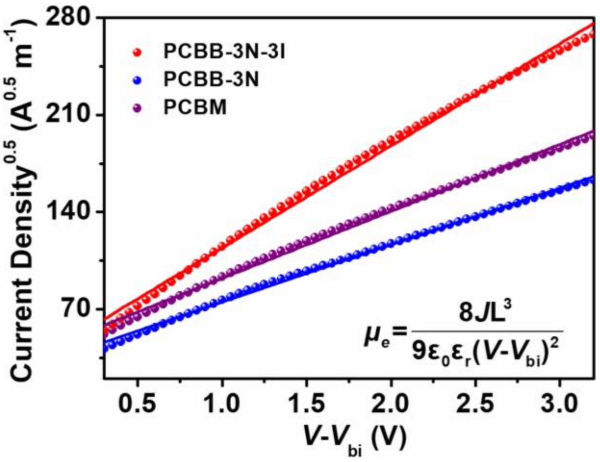

Supplementary Figure 15. *J*-*V* curves for electron-only devices with structure of ITO/ZnO/PCBM or PCBB-3N or PCBB-3N-3I/Al. The mobility μ_e is extracted by fitting the *J*-*V* curves using the Mott–Gurney law (inset equation), where ε_r is ~ 3, V_{bi} is the work function difference between ZnO and Al.

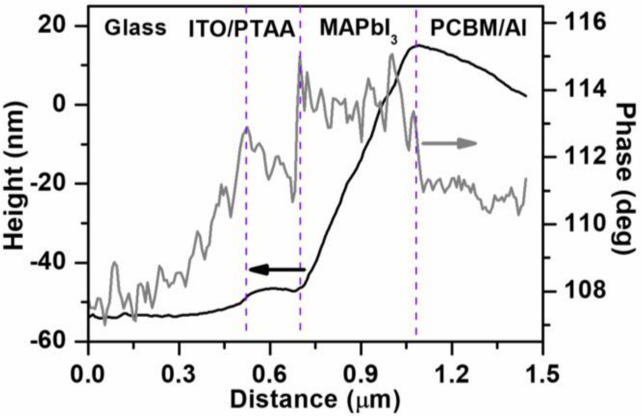

Supplementary Figure 16. Height and phase profiles of control device extracted from Figure 3b-c.

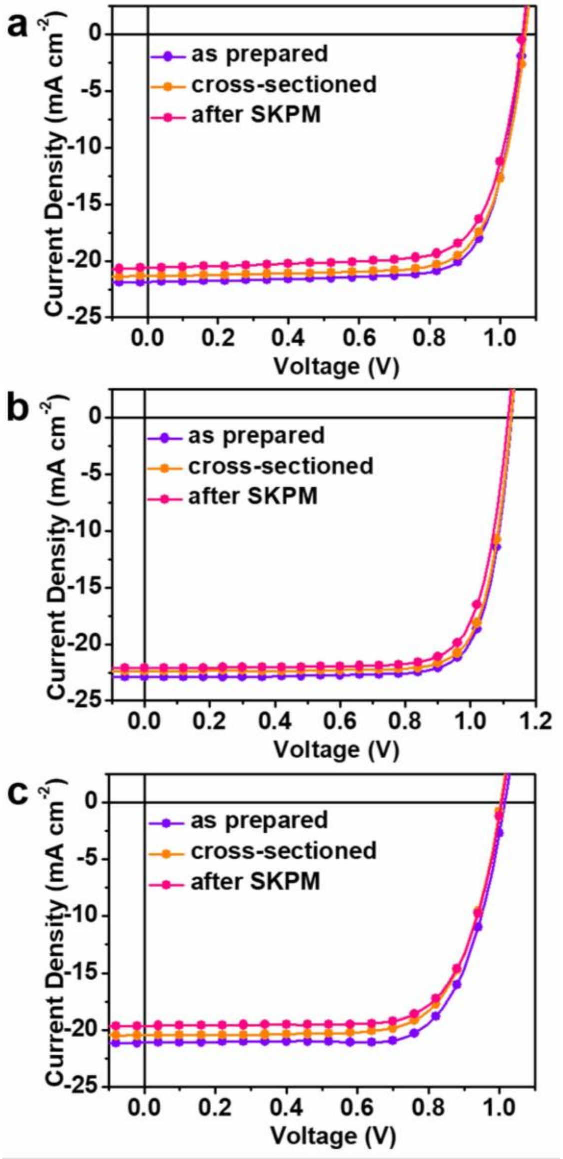

Supplementary Figure 17. *J*-*V* curves of control (a), PCBB-3N-3I (b) and PCBB-3N (c) devices before and after cross-section preparation, and after SKPM measurement.

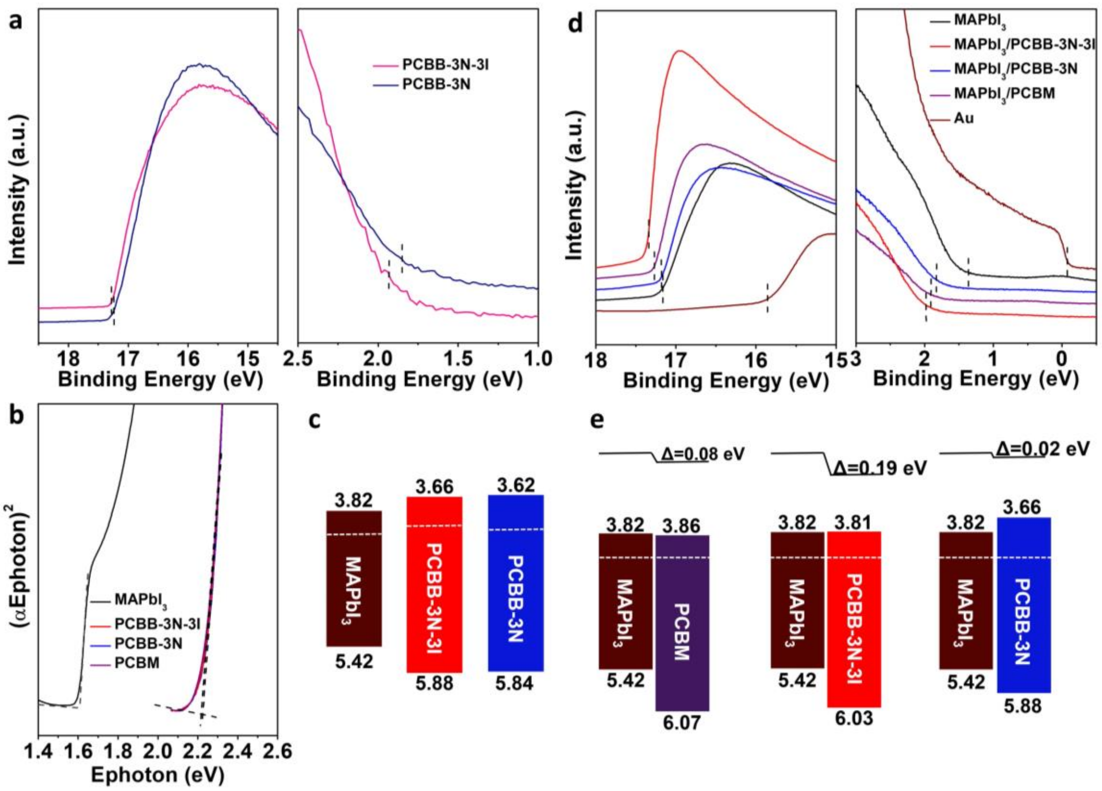

Supplementary Figure 18. (a) UPS spectra of PCBB-3N-3I and PCBB-3N. (b) Tauc-plot of $MAPbI_3$, PCBM, PCBB-3N-3I and PCBB-3N. (c) Energy level diagram of $MAPbI_3$, PCBB-3N-3I and PCBB-3N. (d) UPS spectra of $MAPbI_3$ before and after ultrathin (~ 1 nm) PCBM, PCBB-3N-3I and PCBB-3N treatment. (e) Interfacial energy band structure between $MAPbI_3$ and PCBM/PCBB-3N-3I/PCBB-3N.

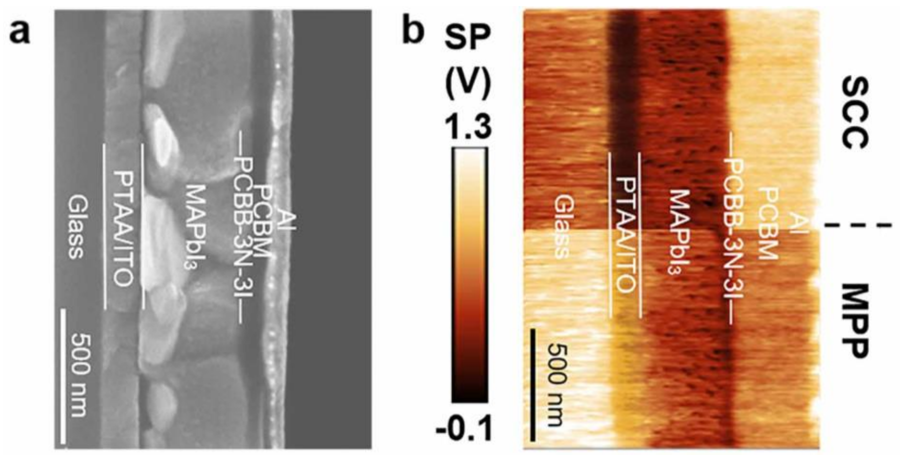

Supplementary Figure 19. SEM image (a) and SKPM measured SP image in SCC (top half) and in MPP (bottom half) (b) of PCBB-3N-3I device cross-section.

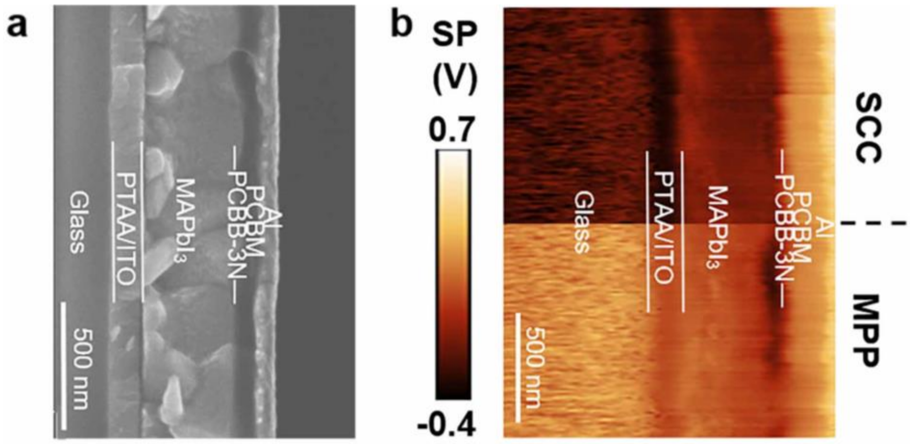

Supplementary Figure 20. SEM image (a) and SKPM measured SP image in SCC (top half) and in MPP (bottom half) (b) of PCBB-3N device cross-section.

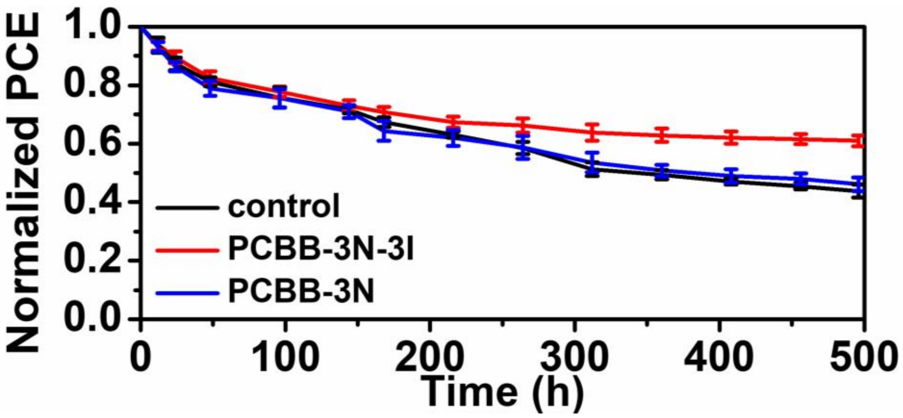

Supplementary Figure 21. Normalized PCE of devices with a structure of ITO/PTAA/$MAPbI_3$/PCBM/C_{60}/BCP/Cu before and after PCBB-3N-3I/PCBB-3N treatment as a function of storage time in ambient condition with 75–85% RH.

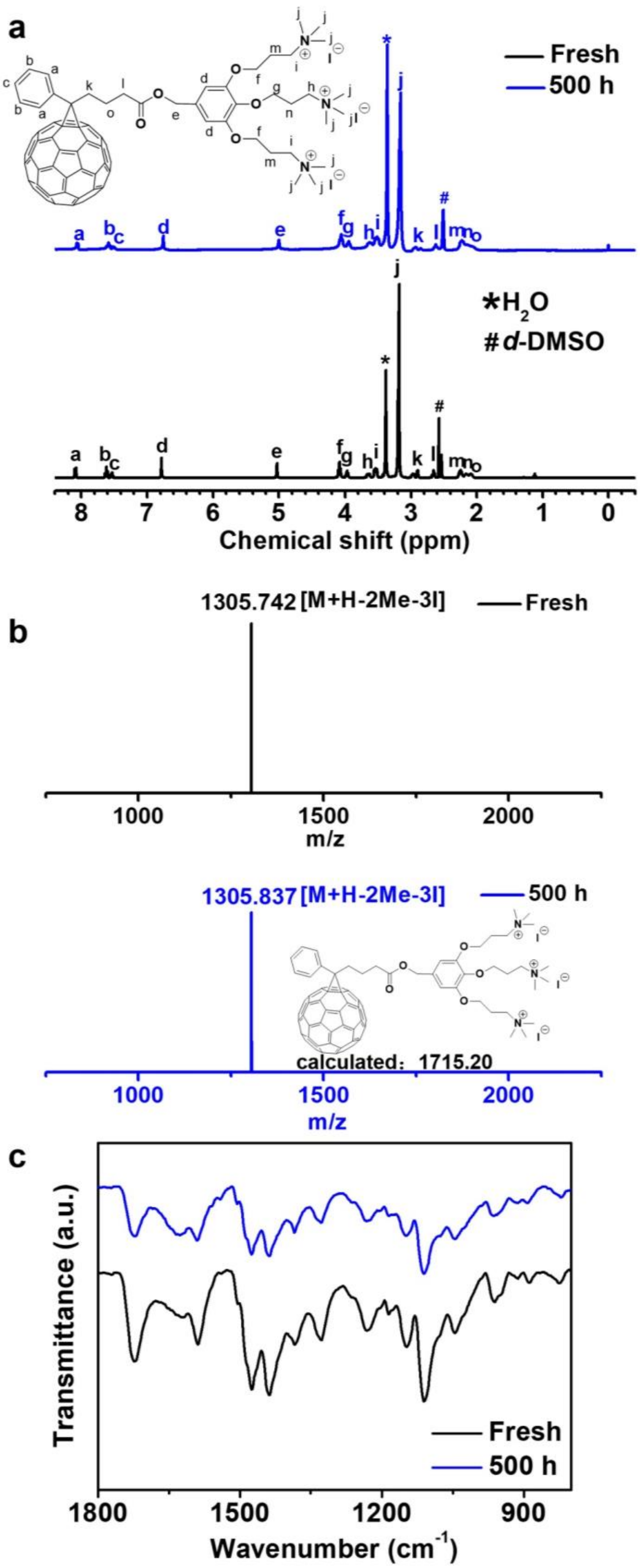

Supplementary Figure 22. Structure characterization of PCBB-3N-3I stored in ambient condition with 75–85% RH. (a) ^{1}H-NMR; (b) TOF-MS; (c) FT-IR.

Supplementary Table 1. Photovoltaic parameters of pero-SCs treated with different concentrations of PCBB-3N-3I

PCBB-3N-3I (mg mL^{-1})	V_{oc} (V)	J_{sc} (mA/cm^2)	*FF* (%)	PCE (%)
0.05	1.104	21.97	74.74	18.13
0.1	1.104	23.20	79.82	20.44
0.2	1.100	22.09	77.36	18.80
0.3	1.075	21.44	76.22	17.57
0.5	1.042	21.21	72.96	16.12

Supplementary Table 2. Photovoltaic parameters of pero-SCs with different concentrations of PCBB-3N

PCBB-3N (mg mL^{-1})	V_{oc} (V)	J_{sc} (mA cm^{-2})	*FF* (%)	PCE (%)
0.05	1.011	21.38	70.69	15.28
0.1	1.025	21.58	70.51	15.60
0.2	1.005	20.70	71.59	14.89
0.3	0.983	20.99	70.67	14.58
0.5	0.967	20.37	61.20	12.06

Supplementary Table 3. A summary of state-of-the-art inverted structure pero-SCs

Perovskite active layer	V_{oc} (V)	J_{sc} (mA cm^{-2})	*FF* (%)	PCE (%)	ref
$FA_{0.85}MA_{0.15}Pb(I_{0.85}Br_{0.15})_3$	1.14	23.7	78	21.0	1
$MAPbI_{3-x}Cl_x$	1.12	22.3	79.8	20.0	2
$MAPbI_3$	1.11	22.96	76.5	19.5	3
$MAPbI_3$	1.03	23.51	83	20.1	4
$MAPbI_3$	1.17	21.80	78	19.9	5
$(FA_{0.95}PbI_{2.95})_{0.85}(MAPbBr_3)_{0.15}$	1.21	22.50	79.0	21.5	6
$MAPbI_3$	1.03	23.74	78	19.2	7
$MAPbI_3$	1.07	23.65	80	20.2	8
$MAPbI_3$	1.115	22.41	79.4	19.9	9
$MAPbI_3$	1.08	22.68	83.4	20.4	10
$Cs_{0.05}FA_{0.81}MA_{0.14}PbI_{2.55}Br_{0.45}$	1.21	22.49	78.5	21.4	11
$Cs_{0.05}(FA_{0.85}MA_{0.85})_{0.95}Pb(I_{0.85}Br_{0.15})_3$	1.12	23.06	80	20.8	12
$MAPbI_3$	1.146	22.69	78.55	20.4	13
$FA_{0.85}MA_{0.15}Pb(I_{0.85}Br_{0.15})_3$	1.15	23.1	81.1	21.5	14
$MAPbI_3$	1.12	22.43	82	20.6	15

Supplementary Table 4. Photovoltaic parameters of control, PCBB-3N-3I and PCBB-3N devices with 0.049 cm^2 mask or w/o mask

	Mask	V_{oc} (V)	J_{sc} (mA cm^{-2})	FF (%)	PCE (%)
control	w/o mask	1.067	22.38	73.33	17.51
	with mask	1.067	22.29	73.79	17.55
PCBB-3N-3I	w/o mask	1.105	23.29	80.86	20.81
	with mask	1.103	23.14	81.45	20.79
PCBB-3N	w/o mask	1.042	20.90	70.41	15.33
	with mask	1.039	20.73	71.06	15.30

Supplementary Table 5. Conductivity and mobility of fullerene derivative

fullerene	conductivity (S cm^{-1})	mobility (cm^2 V^{-1} S^{-1})
PCBM	7.54×10^{-4}	3.99×10^{-4}
PCBB-3N-3I	3.96×10^{-2}	9.24×10^{-4}
PCBB-3N	4.58×10^{-5}	2.90×10^{-4}

Supplementary Table 6. Photovoltaic parameters of control, PCBB-3N-3I, and PCBB-3N devices before and after cross-sectional preparation, and after SKPM measurement

device	stage	V_{oc} (V)	J_{sc} (mA cm^{-2})	FF (%)	PCE (%)
control	as prepared	1.068	21.90	75.63	17.69
	cross-sectioned	1.070	21.29	75.33	17.16
	after SKPM	1.062	20.60	74.37	16.27
PCBB-3N-3I	as prepared	1.125	22.88	79.10	20.36
	cross-sectioned	1.124	22.43	79.17	19.96
	after SKPM	1.115	22.09	77.91	19.19
PCBB-3N	as prepared	1.016	21.13	72.34	15.53
	cross-sectioned	1.004	20.49	71.31	14.67
	after SKPM	1.007	19.63	72.14	14.26

Supplementary Note 1. First-principles density functional theory calculations

Calculation of molecular dipole moment. The optimization of molecular geometry of PCBB-3N-3I and PCBB-3N was performed using the first-principles density functional theory (DFT) method of the three parameter Becke-style hybrid functional (B3LYP) with the basis set of LANL2DZ. All calculations were performed using GAUSSIAN 2009 package.

Calculation of adsorption energy. DFT calculations were performed using the projected augmented wave (PAW).[16] The Perdew-Burke-Ernzerhof exchange-correlation functional of the generalized-gradient approximation (PBE-GGA) was adopted for the exchange-correlation functional.[17] In all calculations, the plane-wave expansion of the wave functions with an energy cutoff of 400 eV was applied. The model of the pristine surface of perovskite used in this work has (3×2×2) supercell and 5-layer atoms. A vacuum region of 20 Å was inserted between the periodic slabs along the Z direction in order to avoid the interlayer interactions. During the geometry optimization, the upper 3 atomic layers of the slab model were allowed to fully relax while the bottom six layer of atoms were fixed at the optimized bulk geometry. During the relaxation, the force tolerance was set to 0.05eV $Å^{-1}$. The adsorption energy (E_{ads}) of small molecule was calculated as follows: $E_{ads} = E_{total} - (E_{sur} + E_{mol})$, where E_{total} is the total energy of adsorption system, E_{sur} is the total energy of surface, E_{mol} is the total energy of small molecule. Based on this equation, the more negative E_{ads} indicates the stronger adsorption strength.

Supplementary Reference

1. Zheng, X. et al. Defect passivation in hybrid perovskite solar cells using quaternary ammonium halide anions and cations. *Nat. Energy* **2**, 17102 (2017).
2. Ye, S. et al. A breakthrough efficiency of 19.9% obtained in inverted perovskite solar cells by using an efficient trap state passivator Cu(thiourea)I. *J. Am. Chem. Soc.* **139**, 7504-7512 (2017).
3. Lin, Y. et al. π-Conjugated lewis base: efficient trap-passivation and charge-extraction for hybrid perovskite solar cells. *Adv. Mater*. **29**, 1604545 (2017).
4. Chiang, C.-H., Nazeeruddin, M. K., Grätzel, M. & Wu, C.-G. The synergistic effect of H_2O and DMF towards stable and 20% efficiency inverted perovskite solar cells. *Energy Environ. Sci.* **10**, 808-817 (2017).
5. Bai, Y. et al. Dimensional engineering of a graded 3D–2D halide perovskite interface enables ultrahigh V_{oc} enhanced stability in the p-i-n photovoltaics. *Adv. Energy Mater.* **7**, 1701038 (2017).
6. Luo, D. et al. Enhanced photovoltage for inverted planar heterojunction perovskite solar cells. *Science* **360**, 1442 (2018).
7. Ok, S. A. et al. Management of transition dipoles in organic hole-transporting materials under solar irradiation for perovskite solar cells. *Nat. Commun.* **9**, 4537 (2018).
8. Xu, G. et al. New strategy for two-step sequential deposition: incorporation of hydrophilic fullerene in second precursor for high-performance p-i-n planar perovskite solar cells. *Adv. Energy Mater.* **8**, 1703054 (2018).
9. Ma, J. et al. Efficient and stable nonfullerene-graded heterojunction inverted perovskite solar cells with inorganic Ga_2O_3 tunneling protective nanolayer. *Adv. Func. Mater.* **28**, 1804128 (2018).
10. Meng, X. et al. Molecular design enabled reduction of interface trap density affords highly efficient and stable perovskite solar cells with over 83% fill factor. *Nano Energy* **52**, 300-306 (2018).
11. Yang, S. et al. Tailoring passivation molecular structures for extremely small open-circuit voltage loss in perovskite solar cells. *J. Am. Chem. Soc.* **141**, 5781-5787 (2019).
12. Liu, X. et al. 20.7% Highly reproducible inverted planar perovskite solar cells with enhanced fill factor and eliminated hysteresis. *Energy Environ. Sci.* **12**, 1622-1633 (2019).
13. Wu, T. et al. Efficient defect passivation for perovskite solar cells by controlling the electron density distribution of donor-π-acceptor molecules. *Adv. Energy Mater.* **9**, 1803766 (2019).
14. Bai, Y. et al. Oligomeric silica-wrapped perovskites enable synchronous defect passivation and grain stabilization for efficient and stable perovskite photovoltaics. *ACS Energy Lett.* **4**, 1231-1240 (2019).

15. Yang, D. et al. Stable efficiency exceeding 20.6% for inverted perovskite solar cells through polymer-optimized PCBM electron-transport layers. *Nano Lett.* **19**, 3313-3320 (2019).
16. Taylor, J., Brandbyge, M. & Stokbro, K. Theory of rectification in tour wires: the role of electrode coupling. *Phys. Rev. Lett.* **89**, 138301 (2002).
17. Perdew, J, P., Burke, K. & Ernzerhof, M. Generalized gradient approximation made simple. *Phys. Rev. Lett.* **77**, 3865-3868 (1996).
