# Supplementary material for: Reconfiguration of interfacial energy band structure for high-performance inverted structure perovskite solar cells
Source: Nat Commun. 2019 Oct 9;10:4593. doi: 10.1038/s41467-019-12613-8 (PMC6785549; doi:10.1038/s41467-019-12613-8)
Supplement: Supplementary file 3 — Reporting Summary [file 41467_2019_12613_MOESM3_ESM.pdf]

nature research

Corresponding author(s): Qi Chen, Yaowen Li

Solar Cells Reporting Summary

Nature Research wishes to improve the reproducibility of the work that we publish. This form is intended for publication with all accepted papers reporting the characterization of photovoltaic devices and provides structure for consistency and transparency in reporting. Some list items might not apply to an individual manuscript, but all fields must be completed for clarity.

For further information on Nature Research policies, including our data availability policy, see Authors & Referees.

Experimental design

Please check: are the following details reported in the manuscript?

1. Dimensions

Area of the tested solar cells
- [x] Yes
- [ ] No

Methods section: 0.1 cm2.

Method used to determine the device area
- [x] Yes
- [ ] No

Methods section: Device area is defined by cross-overlap of the 0.2 cm wide patterned ITO bar and the 0.5 cm wide Al/Cu bar deposited through a shadow mask.

2. Current-voltage characterization

Current density-voltage (J-V) plots in both forward and backward direction
- [x] Yes
- [ ] No

Fig. 1b

Voltage scan conditions
For instance: scan direction, speed, dwell times
- [x] Yes
- [ ] No

Main text: the voltage was scanned both in forward direction from -0.2 V to 1.2 V and reverse direction from 1.2 V to -0.2 V with a scan speed 0.02 V/s

Test environment
For instance: characterization temperature, in air or in glove box
- [x] Yes
- [ ] No

Main text: in N2-filled glove box

Protocol for preconditioning of the device before its characterization
- [ ] Yes
- [x] No

Main text: no preconditioning

Stability of the J-V characteristic
Verified with time evolution of the maximum power point or with the photocurrent at maximum power point; see ref. 7 for details.
- [x] Yes
- [ ] No

Supplementary Fig. 3: steady-state PCEs were obtained by tracking the maximum power point.

3. Hysteresis or any other unusual behaviour

Description of the unusual behaviour observed during the characterization
- [ ] Yes
- [x] No

Main text: negligible hysteresis and no other unusual behavior

Related experimental data
- [ ] Yes
- [x] No

Not relevant

4. Efficiency

External quantum efficiency (EQE) or incident photons to current efficiency (IPCE)
- [x] Yes
- [ ] No

Fig. 1d.

A comparison between the integrated response under the standard reference spectrum and the response measure under the simulator
- [x] Yes
- [ ] No

Main text: the integrated Jsc obtained from the EQE spectra agrees well with the Jsc value obtained from the J–V curves under the simulator within 5% deviation

For tandem solar cells, the bias illumination and bias voltage used for each subcell
- [ ] Yes
- [x] No

Not relevant.

5. Calibration

Light source and reference cell or sensor used for the characterization
- [x] Yes
- [ ] No

Methods section: light source: a SS-F5-3A solar simulator (Enli Technology CO., Ltd.) for AM 1.5G illumination (100 mW cm-2); reference cell: a standard Si solar cell (SRC-00036)

Confirmation that the reference cell was calibrated and certified
- [x] Yes
- [ ] No

Methods section: the reference cell is calibrated and certified by Enli Technology

Calculation of spectral mismatch between the reference cell and the devices under test

- [x] Yes
- [ ] No

1.017

6. Mask/aperture

Size of the mask/aperture used during testing

- [x] Yes
- [ ] No

Methods section:non-reflective mask with area of 0.049 cm2 was also used to define the cell area

Variation of the measured short-circuit current density with the mask/aperture area

- [ ] Yes
- [x] No

Not relevent

7. Performance certification

Identity of the independent certification laboratory that confirmed the photovoltaic performance

- [ ] Yes
- [x] No

The photovoltaic performance of our devices was not confirmed from independent certification laboratories

A copy of any certificate(s)
Provide in Supplementary Information

- [ ] Yes
- [x] No

Not relevent

8. Statistics

Number of solar cells tested

- [x] Yes
- [ ] No

Main text: more than 20 devices for each kind of devices were tested

Statistical analysis of the device performance

- [x] Yes
- [ ] No

Fig. 1c, Table 1

9. Long-term stability analysis

Type of analysis, bias conditions and environmental conditions
For instance: illumination type, temperature, atmosphere humidity, encapsulation method, preconditioning temperature

- [x] Yes
- [ ] No

Fig. 5a: a shelf-life test in ambient with 40% - 50% and 75% - 85% RH without any encapsulation
